# Targeting the MAT2A-SAM axis increases platinum sensitivity in ovarian cancer by impairing DNA repair and stemness

**DOI:** 10.64898/2026.09.22.753550

**Published:** 2026-10-13

**Authors:** Shu Zhang, Zhen Fu, Yanchi Zhou, Tara X. Metcalfe, Truc T. Vuong, Lindsay W. Brubaker, Benjamin G. Bitler, Heather M. O’Hagan, Kenneth P. Nephew

## Abstract

Metabolic and epigenetic reprogramming drives platinum resistance and recurrence in high-grade serous ovarian cancer (HGSC), presenting a critical clinical challenge. Acquired platinum resistance has been linked to altered DNA methylation, a process requiring S-adenosylmethionine (SAM), synthesized by methionine adenosyltransferase 2A (MAT2A) from methionine metabolism. However, how the MAT2A-SAM axis coordinates metabolic-epigenetic remodeling in HGSC remains unclear. We demonstrate that MAT2A-driven SAM synthesis is essential for platinum-induced DNA methylation dynamics and ovarian cancer stem cells (OCSCs) enrichment. High MAT2A expression correlates with poor progression-free survival in HGSC patients, while genetic knockdown or pharmacological inhibition of MAT2A increases cisplatin sensitivity in HGSC cells. Genome-wide DNA methylation profiling showed that cisplatin reshaped the methylome with prominent hypermethylation at promoter regions, which was reversed by MAT2A knockdown. Transcription factors predicted from differential methylation were enriched in platinum resistance, DNA repair, and stemness pathways. Mechanistically, disrupting MAT2A-SAM axis suppresses a novel SAMTOR-mTOR-S6K-FANCD2 signaling pathway. This blockade leads to the accumulation of R-loops, attenuates both homologous recombination (HR) and non-homologous end joining (NHEJ) repair pathways, and enhanced platinum-induced cell death. Furthermore, transcriptomic analysis of paired primary and recurrent HGSC tumors links MAT2A increase to elevated OCSC features during recurrence. Consistently, MAT2A inhibition prevents cisplatin-increased OCSC population and sphere-forming capacity in vitro, both of which are rescued by exogenous SAM supplementation. Together, our findings identify the MAT2A-SAM axis as a pivotal metabolic-epigenetic regulator of the DNA damage response and cancer stemness, establishing MAT2A inhibition as a therapeutic strategy to enhance platinum sensitivity in HGSC.

**Highlights:**

- Cisplatin remodels the HGSC cell methylome with predominant promoter hypermethylation
- MAT2A-SAM is essential for platinum-induced methylome remodeling and OCSC enrichment
- MAT2A loss impairs activation of HR and NHEJ repair via mTOR-S6K-FANCD2 signaling
- MAT2A inhibition increases platinum sensitivity in both MTAP-proficient and -deficient HGSC cells tested

## Full Text Availability

The license terms selected by the author(s) for this preprint version do not permit archiving in PMC. The full text is available from the preprint server.

